# Preventive health behaviours during coronavirus disease 2019 pandemic based on health belief model among Egyptians

**DOI:** 10.1186/s43045-020-00051-y

**Published:** 2020-10-06

**Authors:** Ayah M. Barakat, Zeinab A. Kasemy

**Affiliations:** 1 Department of Family Medicine, Menoufia Faculty of Medicine, Menoufia University, Menoufia, Egypt; 2 Department of Public Health and Community Medicine, Menoufia Faculty of Medicine, Menoufia University, Menoufia, Egypt

**Keywords:** Preventive health behaviours, COVID-19, Health belief model

## Abstract

**Background:**

Coronavirus disease 2019 (COVID-19) is spreading rapidly in the world, and on 11 March 2020, WHO announced the outbreak a global pandemic. Given the severity of this major outbreak and the importance of prevention and protection against the spread of SARS-CoV-2, the predictors of engaging in the preventive behaviours could potentially be of great practical importance as it could help us identify high-risk groups and take the necessary steps towards improving their health behaviour. As the health behavioural response of the Egyptian population during COVID-19 is unknown and the health belief model constructs can be used to explain health behaviour, this study was conducted to assess the preventive behaviours to COVID-19 and the associated role of health belief model constructs over three periods of time; at the beginning of COVID-19 pandemic then 4 weeks and10 weeks later.

**Results:**

Perceived severity and benefits of health belief model constructs showed significant decrease in the 2^nd^ interview, followed by an increase in the 3^rd^ interview (*P* < 0.001). Perceived barriers showed a significant increase in the 2^nd^ interview followed by a significant decrease in the 3^rd^ interview (*P* < 0.001). Knowledge score was significantly lower at the start, then a surge happened in the next interview followed by a slight drop in the 3^rd^ interview (*P* < 0.001). Preventive behaviours were significantly lower in the 2^nd^ interview then significantly higher in the 3^rd^ interview (*P* < 0.001). On the analysis of the factors associated with preventive behaviours using multivariate regression, the results determined age, high education, being a health care worker, perceived susceptibility, benefits, barriers and self-efficacy.

**Conclusions:**

Perceptions of benefits could be increased by tailoring communication strategies to various groups, emphasizing how different people can engage in effective preventive behaviours. Policy makers should pay attention to lower-educated persons living in rural areas being a group with the least engagement in health-protective actions.

## Background

Coronaviruses (CoV) are a large family of viruses that can affect both humans and animals (cattle, camels, cats and bats). Sometimes humans can be infected by animal coronaviruses, such as severe acute respiratory syndrome coronavirus (SARS-CoV), Middle East respiratory syndrome coronavirus (MERS-CoV) and now Severe acute respiratory syndrome coronavirus 2 (SARS-CoV-2). Acute respiratory tract infection due to SARSCoV-2, now officially called the novel coronavirus disease (coronavirus disease 2019 (COVID-19)), is currently spreading rapidly worldwide and has become a public health concern [1]. COVID-19 is characterized by high fever, difficult breathing, dry cough and atypical pneumonia and is usually confirmed by positive RNA test or computed tomography of the lungs [2]. COVID-19 is spreading rapidly in the world, and on 11 March 2020, WHO announced the outbreak a global pandemic [3]. After the first major outbreak in Wuhan, China in January, South Korea, Iran and Italy in late February and early March 2020 became involved in this pandemic [4]. Many countries are currently combating COVID-19 and trying to prevent its further spread and reduce morbidity and mortality rates, and to thereby diminish the overall stress and tension in their healthcare system [5]. Since there is no definitive cure and specific vaccine for SARS-CoV-2, all measures to supplying public health rely on preventing the spread of the virus by droplets, close contact and contaminated surfaces [1]. The prevention and control of this disease by observing the rules of prevention and personal hygiene, such as washing hands regularly with soap and water and covering mouth and nose when coughing and sneezing, are the only ways to deal with this disease. Each individual is the most important factor in preventing the disease and maintaining health; and the right or wrong behaviours are influenced by the individuals’ beliefs, values, and habits [6]. Sociologists and psychologists have constructed different theories and models to explain the factors affecting the health behaviour. The health belief model (HBM) is one of this models which is introduced by Rosenstock et al. [7] and is a theoretical guideline for health behaviours in the public health research. The health belief model is popular and generally acceptable because it has a high predictive power. The history of the health belief model (HBM) dates back to the 1950s when a group of public health social psychologists were attempting to understand why the general public failed to utilize free county screening programs aimed at early detection of asymptomatic diseases such as tuberculosis [8]. The main constructs of the model include the following four perceptions: perceived seriousness, perceived susceptibility, perceived benefits and perceived barriers. Each of these perceptions can be used to explain health behaviour. Other constructs have been added to the HBM; thus, the model has been expanded to include cues to action and self-efficacy [9]. Perceived susceptibility posits that the more an individual perceives the risk of a disease, the more likely he or she will change behaviour to diminish that risk [10]. Perceived severity includes some evaluation of the consequences of a disease based on knowledge and some beliefs about the bad effect of a certain behaviour or disease that might affect an individual. Perceived benefit, the third dimension, suggests that individuals perceive the value and usefulness of adopting new behaviours in regard to minimizing the risk of an illness and will likely adopt new behaviours based on their perceptions of their benefits in reducing threats [9]. The fourth dimension, perceived barrier, is the most powerful dimension of HBM, in that individuals evaluate the obstacles and difficulties they might face when adopting a new behaviour. This dimension, however, might result in individuals abandoning a new behaviour [11]. The health behavioural responses of the Egyptian population during COVID-19 are still unknown. Given the severity of this major outbreak and the importance of prevention and protection against the spread of COVID-19, we carried out the present study to identify the preventive health behaviours from COVID-19 based on the health belief model among Egyptian population and to explore the predictors of engaging in these behaviours. These predictors could potentially be of great practical importance as it could help us identify high-risk groups and take the necessary steps towards improving their health behaviour.

## Methods

For 3 months, a cross-sectional study was carried out. The data were collected using personal interviews in addition to social networking sites and cell phone calls. Persons who were Egyptians, aged 18 years or more, and agreed to participate in the study, after the explanation of objectives, procedures, voluntary nature of participation and declarations of confidentiality, were asked to complete a semi-structured questionnaire. The participants were asked to answer the questionnaire three times at the beginning of pandemic and 4 weeks and 10 weeks later. On 14^th^ of February, the first case in Egypt involving a Chinese person was announced. The first time of data collection started 3 weeks later from 7^th^ of March to 14^th^ of March. During the first time, the data were collected through personal interviews in governmental settings and houses also through the social network using Google forms. There was a plan number to reach a sample size of 377 participants as the probability to occur equals probability of not to occur (*p* = *q* = 50%). The collected data included 380 questionnaires (220 personal interviews and 160 online questionnaires). The second time of data collection began about 4 weeks after the first time (from 14^th^ of April to 21^st^ of April). The collected data included 210 questionnaires (150 personal interviews and 60 online questionnaires using emails of the participants shared in the first time). The third time was nearly 10 weeks later from 23^rd^ May to 30^th^ of May. The collected data included 182 questionnaires (150 personal interviews and 32 online questionnaires). The time required to complete the questionnaire was about 12 min. The number of persons who answered the questionnaire was 380, 210 and 182 at the first, second and third times, respectively. The number decreased each time due to the dropout of the participants. So, the final total number involved in the study was 182. A pilot study on ten participants (about 6%) was applied to validate the questionnaire. Experts in public health and family medicine specialties measured the questionnaire relevancy and ability to correctly figure out preventive behaviours and perceptions of Egyptians regarding COVID-19 based on the HBM. Previous surveys on the health preventive behaviours and the health belief model were reviewed. Additional questions about COVID-19 were added. The questionnaire has a good reliability as Cronbach’s alpha has been calculated, and it was 0.83. The participants were subjected to four questionnaires titled (I) Socio-demographic characteristic (II) COVID-19 specific knowledge (III) Health belief model constructs and (IV) Health preventive behaviours from COVID-19. In (I) Socio-demographic characteristics, socioeconomic standard was assessed according to Fahmy et al. [12]. The knowledge questionnaire about COVID-19 (II, score ranges from 12 to 24) has twelve questions: clinical presentations (4 questions), transmission routes (3 questions), and prevention and control of COVID-19 (5 questions) [13]. Adequate knowledge was reported if participants scored ≥ 60%. The Health belief model constructs questionnaire (III) involves six sections: a. perceived susceptibility (4 questions; (a) Feeling more likely to contract the disease, (b) Having close contact with or knowing any individuals affected with COVID-19, (c) Having any COVID-19 symptoms and (d) Caring about this disease and changing daily activities than before); b. perceived severity (4 questions; the degree to which (i) the disease has a high mortality rate, (ii) the disease can cause severe and serious symptoms, (iii) the disease appears to spread easily among people and (iv) difficulty in treatment of symptoms present); c. perceived benefits (2 questions; (a) Regular hand washing can easily decrease the risk of being infected by the disease, (b) Prevention of disease by personal protective equipment); d. perceived barriers (8 questions; (i) Preventive instructions are difficult to be done, (ii) Not having the patience to continue following the preventative instructions, (iii) The regular hand washing with soap and water as a boring task, (iv) The mask is scarce in the market, (v) Disinfectant gels and solutions are rare and expensive in the market, (vi) Alcohol pads are difficult to get in the market, (vii) Not touching hands, mouth, nose and eyes being a difficult task and (viii) Difficulty in staying at home to prevent the disease; e. perceived sense of self-efficacy (1 question; asking respondents about the ability to follow preventive instructions against the disease); and f. cues to action (3 questions; (a) TV, radio and internet information about the disease were helpful, (b) the local government encouraged them to follow preventive behaviour for the disease scales and (c) the family members encouraged them to follow preventive behaviour for the disease). HBM questions used a 5-point Likert scale (from strongly agree to strongly disagree), with a range from 1 to 5 except for perceived susceptibility has yes/ no response. The higher the total scores are, the higher the susceptibility to be infected with COVID-19 disease will be. In the Health preventive behaviours to COVID-19 questionnaire (IV) assessment was done using 8 items (ability to (i) bend elbow or place a tissue paper in front of the mouth and nose when sneezing or coughing, (ii) keep a distance of at least one meter from others, (iii) not kiss or shake hands of others (iv) not leave the house unless absolutely necessary, (v) wash hands with soap and water regularly for at least 20 s, (vi) not touch eyes, mouth and nose,(vii) not get cell phone out of pocket, and (viii) wash hands with soap and water immediately after getting in home. A 5-point Likert scale from Always to Never, with range from 1 to 5 is applied.

### Ethical considerations

This work was approved by our institutional research board. Respondent’s confidentiality was ensured. A consent form appended to the online questionnaire. By clicking the ‘Begin survey’ link, the respondent indicated his/her consent to participate in the study and the informed consent was electronically recorded. Written informed consent was signed by the participants of personal interview.

### Statistical analysis

Results were statistically analyzed by SPSS version 22 (SPSS Inc., Chicago, IL, USA). The non-parametric data were analyzed using Mann–Whitney and Friedman tests. Regression analysis was used to detect the predictors of an independent variable. *P* value < 0.05 is considered significant.

## Results

**Fig. 1:**
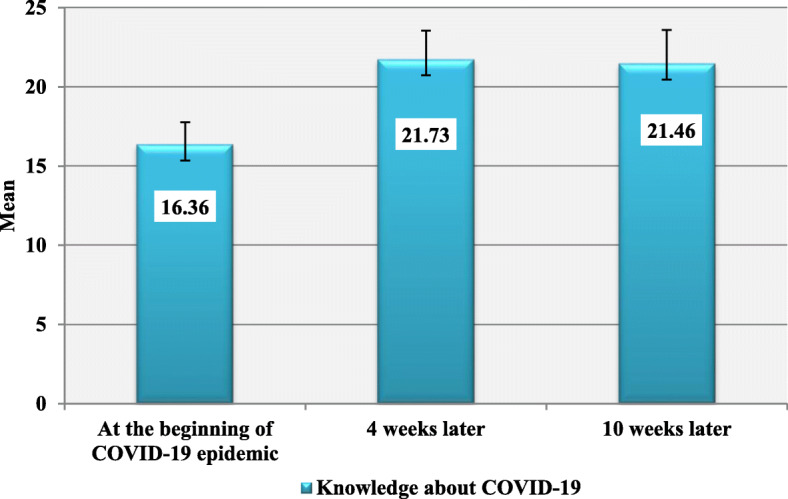
Distribution of total knowledge about COVID 19 among participants over the three periods of time

About two thirds of 182 participants were females, of rural residence, non-healthcare workers and with moderate socio-economic standard (Table 1). The study was conducted at the beginning of COVID-19 pandemic and 4 weeks and 10 weeks later. There were significant differences between the three measurements of the HBM regarding all items including perceived susceptibility, severity, barriers, benefits, ability to follow preventive instructions and cues to action (*P* < 0.001)**.** Perceived susceptibility showed an increase in the scores, while ability to follow preventive instructions and cues showed decreases in the scores over the three interview periods (*P* < 0.001). Perceived severity and benefits showed decrease in the 2^nd^ interview (17.02 ± 2.61 and 8.43 ± 1.50, respectively), followed by an increase in the 3^rd^ interview (17.90 ± 2.2 and 8.79 ± 1.31, respectively) (*P* < 0.001). Perceived barriers showed an increase in the 2^nd^ interview (31.51 ± 5.37) followed by a decrease in the 3^rd^ interview (28.81 ± 4.99) (*P* < 0.001) (Table 2). Knowledge scores were significantly lower at the start (16.36 ± 1.42), then a surge happened in the next interview (21.73 ± 1.81) followed by a slight drop in the 3^rd^ interview (21.46 ± 2.13) (*P* < 0.001) (Fig. 1). The three interviews of observing preventive behaviours from COVID-19 at the beginning of the pandemic and 4 weeks and 10 weeks later, showed significant difference where it was significantly lower in the 2^nd^ interview (24.99 ± 3.18) then significantly higher in the 3^rd^ interview (31.03 ± 3.19) (*P* < 0.001) (Table 3). Multivariate regression showed that age; high education; being a healthcare worker (HCW); perceived susceptibility, benefits, and barriers; and ability to follow the preventive measures against the disease were associated with COVID-19 preventive behaviours, while cues to action, sex and residence variables lost their significance. Perceived susceptibility and perceived benefits had positive relationships. The perceived barriers and age had negative relationships. Women had a higher mean score than men, and also, the mean score was higher in urban residents than rural ones. HCWs reported the highest impact (*β* = 0.38). The perceived benefits (*β* = 0.239) and susceptibility (*β* = 0.162) variables had a higher effect on preventive behaviours than self-efficacy (*β* = 0.158) (Table 4).

**Table 1:** Characteristics of the studied participants

	*N* = 182	
	No.	%
Age (years)		
Mean ± SD	30.19 ± 9.49	
Range	18–67	
Sex		
Male	63	34.6
Female	119	65.4
Residence		
Rural	113	62.1
Urban	69	37.9
Education		
Basic	11	6.0
Secondary	27	14.8
University	111	61.0
Post-graduate	33	18.1
Marital status		
Single	40	22.0
Married	142	78.0
Occupation		
HCWs	69	37.9
Non-HCWs	113	62.1
Socioeconomic standard		
Low	28	15.4
Moderate	119	65.4
High	35	19.2

*SD* standard deviation, *HCWs* health care workers

**Table 2:** Distribution of health belief model constructs for COVID-19 preventive behaviours over three periods of time

	At the beginning of pandemic	4 Weeks later	10 Weeks later	Test of sig *P* value	Post hoc test
	Mean ± SD	Mean ± SD	Mean ± SD		
Perceived susceptibility	5.45 ± 0.76 5 (5–6)	5.59 ± 0.82 6 (5–6)	5.91 ± 0.89 6 (5–6)	102.96 *P* < 0.001*	*P*_1,2,3_ < 0.001*
Perceived severity	17.09 ± 2.62 18(15–19)	17.02 ± 2.61 18 (15–19)	17.90 ± 2.22 18 (17–20)	97.78 *P* < 0.001*	*P*_1_ = 0.010* *P*_2,3_ < 0.001*
Perceived barriers	26.85 ± 5.75 27 (23.75–31.25)	31.51 ± 5.37 32 (29–36)	28.81 ± 4.99 29 (27–32)	190.63 *P* < 0.001*	*P*_1,2,3_ < 0.001*
Perceived benefits	8.58 ± 1.44 9 (8–10)	8.43 ± 1.50 9 (8–10)	8.79 ± 1.31 9 (8–10)	14.77 *P* = 0.001*	*P*_1_ = 0.378 *P*_2_ = 0.062 *P*_3_ < 0.001*
Perceived self-efficacy	3.93 ± 0.99 4 (4–5)	3.45 ± 1.13 4 (3–4)	3.79 ± 1.01 4 (3–4)	102.91 *P* < 0.001*	P_1,2,3_ < 0.001*
Cues to action	11.32 ± 1.46 11 (10–12)	10.18 ± 1.66 10 (9–11)	9.72 ± 1.89 9 (8–11)	149.54 *P* < 0.001*	*P*_1,2,3_ < 0.001*

*Significant, data are expressed as mean ± SD, median (IQR)

**Table 3:** Distribution of preventive behaviours from COVID-19 over the three periods of time

	At the beginning of pandemic	4 Weeks later	10 Weeks later	Test of sig *P* value	Post hoc test
	Mean ±SD	Mean ± SD	Mean ± SD		
Cough and respiratory etiquette	4.08 ± 0.99 4 (4–5)	3.68 ± 0.85 4 (3–4)	4.29 ± 0.77 4 (4–5)	93.05 *P* < 0.001*	*P*_1,2,3_ < 0.001*
Social distancing of at least 1 m	3.36 ± 0.91 3 (3–4)	3.31 ± 0.79 3 (3–4)	4.21 ± 0.69 4 (4–5)	188.79 *P* < 0.001*	*P*_1_ = 0.409 *P*_2,3_ < 0.001*
No shaking hands or kissing others	3.41 ± 1.18 3 (3–4)	3.21 ± 0.87 3 (3–4)	4.20 ± 0.67 4 (4–5)	174.63 *P* < 0.001*	*P*_1_ = 0.008 *P*_2,3_ < 0.001*
Leaving house for absolutely necessary things	3.61 ± 1.01 3 (3–4)	3.15 ± 0.73 3 (3–4)	4.17 ± 0.74 4 (4–5)	186.99 *P* < 0.001*	*P*_1,2,3_ < 0.001*
Washing hands regularly with soap and water for at least 20 s	3.15 ± 0.73 4 (3–4)	4.17 ± 0.74 3 (3–3)	3.62 ± 0.75 4 (3–4)	113.90 *P* < 0.001*	*P*_1,3_ < 0.001* *P*_2_ = 0.862
Not touching eyes, nose and mouth by hands	3.29 ± 0.83 3 (3–4)	2.91 ± 0.55 3 (3–3)	3.39 ± 0.61 3 (3–4)	87.48 *P* < 0.001*	*P*_1,3_ < 0.001* *P*_2_ = 0.049
No taking cell phone out of pocket	2.93 ± 1.10 3 (2–4)	2.68 ± 0.92 3 (2–3)	3.35 ± 1.04 3 (3–4)	145.10 *P* < 0.001*	*P*_1,2,3_ < 0.001*
Washing hands with soap immediately after entering home and before touching any things	3.15 ± 0.98 3 (2–4)	2.90 ± 0.89 3 (2–4)	3.77 ± 0.94 4 (3–4)	214.46 *P* < 0.001*	*P*_1,2,3_ < 0.001*
Total	27.44 ± 4.28 28 (25–31)	24.99 ± 3.18 25 (23–27)	31.03 ± 3.19 31.5 (29–33)	298.72 *P* < 0.001*	*P*_1,2,3_ < 0.001*

*Significant, data are expressed as mean ±SD, median (IQR)

**Table 4:** Effects of health belief model constructs, total knowledge and demographic variables on COVID-19 preventive behaviours during the beginning of pandemic (first time of data collection)

	Standardized coefficients	*P* value	95.0% CI	
			Lower bound	Upper bound
Age	− 0.119	0.024*	− 0.101	− 0.007
Sex (male vs. female)	0.031	0.547	− 0.637	1.199
Residence (rural vs. urban)	0.087	0.107	− 0.166	1.694
Education (low vs. high)	0.148	0.009*	0.395	2.724
Occupation (HCWs vs. others)	0.380	< 0.001*	2.381	4.328
Socioeconomic standard (Low to middle vs. high)	0.042	0.403	− 0.623	1.543
Perceived susceptibility 1	0.162	0.004*	0.303	1.526
Perceived severity 1	0.002	0.978	− 0.184	0.190
Perceived barriers 1	− 0.131	0.013*	− 0.175	− .021
Perceived benefits 1	0.239	< 0.001*	0.356	1.067
Perceived self-efficacy 1	0.158	0.005*	0.207	1.158
Cues to action 1	− 0.001	0.979	− 0.307	0.299
Total knowledge 1	0.065	0.227	− 0.122	0.511

* Significant, *R*^2^ = 0.614, adjusted *R*^2^ = 0.584

^1^Data obtained from result of first time of data collection

## Discussion

The aim of the present study was to determine the preventive behaviours from COVID-19 over three periods of time—at the beginning of COVID-19 pandemic and 4 weeks and 10 weeks later—and roles of health belief model constructs in the disease in Egypt. The results indicated that adherence to preventive behaviours from COVID-19 was at an undesirable low level over the three periods. It is different from a study conducted in the USA which indicated that self-reported compliance was relatively high, and most people indicated that they were nearly always compliant with COVID-19 mitigation measures [14]. This undesirable level of adherence at the beginning of the pandemic may be justified by the result of a study done by Bish and Michie [15] which showed that while confidence in governments, health professionals and medical services has been reported as crucial for individuals to adopt behavioural change, it is possible that overconfidence results in reckless behaviour, as it is assumed that everything will be under control no matter what individual actions are performed. This finding points at a dilemma, as confidence in authorities is needed to establish protective behaviour in the first place and reduce panicking or intense fear of the outbreak [16]. Health communications therefore need to highlight the importance of the individual’s actions as part of the greater societal outcomes, and simultaneously communicate conviction in recommended measures and risk. Further, the decrease in behaviour measures suggests that behaviour changes might sometimes be counterintuitive. This may be due to the ongoing nature of the threat, the requirement to consistently engage in sometimes complex and unpleasant behaviours over a long period of time, and information or media fatigue resulting in reduced behavioural engagement. Also, an Australian study conducted in an earlier stage of the pandemic by Faasse and Newby [17] found positive predictions by media exposure, concern and worry, as well as effectiveness of behaviour. In addition, confidence in authorities and believing that too much fuss was made resulted in less health-protective behaviour [18]. The participants’ knowledge about COVID-19 was poor at the beginning of the pandemic then increased after 4 weeks. This is likely because since the onset of the first confirmed case of the disease, an aggressive media campaign on preventive measures has been started by the Egyptian government to educate the population about these measures and to limit the person-to-person transmission of the virus. There was no relationship between the population’s knowledge and engagement in protective behaviour. As knowledge and media exposure were, on average, quite high. It is similar to a result of the study conducted in Norway, Northern Europe which showed that knowledge had no or even a negative relationship with engagement in protective behaviour [18]. There are fluctuations in the level of preventive behaviours from COVID-19 at different time sequences where they were higher when assessed through the first interview, then decreased through the second interview and re-increased through the third one. There are many factors that could share in these fluctuations, for example, but not limited to, the effect of friends, diseased or deceased friend or relative, and social media which seem to have strong effect on most of its followers. So, the related effects may have a role in increasing or decreasing the preventive behaviors. During the third interview, the morbidity and mortality from the COVID-19 increased suddenly, and the number of diseased and dead persons was announced daily through the social media; as the individuals were affected by the presence of a relative or a friend infected by the virus, the fear was increased again, and the preventive behaviours were followed through this time. The adherence to preventive behaviours against COVID-19 was higher in women than men probably since men have other life concerns than spending time following the preventive measures. Also, women were more motivated for health than men. In studies conducted about women’s behaviour towards breast cancer screening, the health motivation was confirmed as an independent variable. In a study done in Hong Kong on the pandemic of H1N1, women had better performance than men in the prevention of the disease [19]. The performance of preventive behaviours against COVID-19 was higher in urban residents than villagers. Similarly, a study conducted in Northern Iran found that people living in urban areas showed better adherence to the preventive measures against the disease than people living in rural areas; it is probably due to the difference in their educational levels [20]. The preventive behaviours were increased among highly educated persons; the performance of preventive behaviours was increased with increasing educational level. School education increases the knowledge about common health problems and healthy habits as these are included in the standard school curricula. Also, educated persons are more likely to be able to comprehend and understand better what they read. So, they can understand health education messages presented in mass media and through other methods more than the less-educated ones. The less-educated persons had inadequate knowledge about the disease and perceived immortality to the disease. Low education groups also face higher barriers to compliance with social distancing rules, such as staying home or avoiding public transport, due to economic hardship and the fear of losing income. The preventive behaviours were higher in HCWs. It is due to direct contact while dealing with their patients with high perception of susceptibility and having good knowledge about the disease. Perceived barriers were negatively related to the preventive behaviours from COVID-19. Therefore, the rate of adherence to preventive behaviours increased by decreasing perceived barriers. The perceived barriers are important and effective constructs of the health belief model because the individuals should take control over the barriers to behaviour despite their inner desire to engage in preventive behaviour. Excessive barriers can be obstacles and prevent the initiation of desired health behaviours. In the present study, the participants had less perceived barriers to preventive individual behaviours, such as hand washing, but environmental barriers such as shortage of masks, alcohol pads, and disinfectant agents strongly influenced the behaviours. These environmental barriers were more observed on the second time of data collection and were associated with the lowest practice of preventive behaviours through the three measurements. Deficiency of masks has been observed in most regions of the world because of the COVID-19 pandemic, and the issue was also noticed in this study. As, on 29 Mar 2020, Mohamed Ismail, the head of the medical supplies division at the Federation of Egyptian Chambers of Commerce (FECC), announced that there was a shortage of facemasks in the domestic market, and gloves were deficient [21]. A recent study conducted in China showed that the cause of not using the mask was its unavailability in the market [22]. Providing masks and other disinfectants and taking control over the environmental barriers can be effective in increasing the individuals’ compliance to these preventive behaviours. The perception of self-efficacy (ability to follow every preventive instruction against the disease) was associated with preventive behaviours from COVID-19. The existence of increased self-efficacy perception is an important factor in overcoming the perceived barriers, and it was an effective variable for engaging in the preventive behaviours from COVID-19 in the present study. The definition of self-efficacy is the level of confidence in taking control over barriers to a healthy behaviour. According to the health belief model, individuals should have an appropriate level of self-efficacy to overcome barriers to behavior [20]. The more people believe in their ability and skill to attain a certain goal, the more probably they will be able to reach that goal. This study showed that the perceived susceptibility had positive relationships with the performance of preventive behaviour, and it is increased over time. This may be due to the continuous increase in the number of infected cases reported by the Egyptian ministry of health and through time, increase in numbers of infected cases known by the participants (friends, relatives) or participants who had close contact with any individuals affected with the disease. So, respondents felt more likely to contract the disease with increase in perception of susceptibility over time. This study showed that the perceived benefits had positive relationships with the performance of preventive behaviour. So, the individuals perform better by increasing the perceived benefits. Having perceptions such as preventive effect of personal protective equipment use such as disposable gloves and masks and regular hand washing can lead to high perceived benefits, and they are thus strong motivations for following the preventive measures against this disease. Nevertheless, the implication seems to be that motivating people to practice protective behaviour works best by emphasizing that it is effective, rather than by exaggerating risks of not engaging in it.

### Limitations of the study

There was a drop out of participants through the study as the number of participants during the first time of data collection was 380, and in the third time of data collection, we could reach 182 participants only, but this may be due to the main constructs of the study to measure response over 3 time periods. Honest answers were encouraged by ensuring confidentiality of the results, and existing research shows that there can be strong concordance between self-reported and objective compliance measures when surveys are utilized, and this agrees with Dieltjens et al. [23].

## Conclusion

Perceptions of benefits of protective behaviour are crucial. They could be increased by tailoring communication strategies to various groups, emphasizing how different people can engage in effective preventive (hygienic) or avoidance (distancing) behaviour. Policy makers should pay attention to less-educated persons living in rural areas representing a group with the least engagement in health-protective actions.

## Data Availability

Data that support the findings of this study are available from the corresponding author upon reasonable request.

## Abbreviations

COVID-19: Coronavirus disease 2019
WHO: World Health Organization
MERS-CoV: Middle East respiratory syndrome coronavirus
SARS-CoV-2: Severe acute respiratory syndrome coronavirus 2
HBM: Health belief model
SPSS: Statistical Package for the Social Sciences
HCWs: Health care workers
